# Mpox: a review of laboratory detection techniques

**DOI:** 10.1007/s00705-023-05848-w

**Published:** 2023-08-05

**Authors:** Yunfan Zhou, Zixin Chen

**Affiliations:** School of Medicine, Guangzhou Higher Education Mega Centre, South China University of Technology, Panyu District, Guangzhou, 510006 China

## Abstract

Mpox (formerly monkeypox) is a zoonotic disease caused by monkeypox virus (MPXV), which, like smallpox, is characterised by skin rashes. While the world is currently grappling with the coronavirus disease 2019 pandemic, the appearance of MPXV has presented a global threat and raised concerns worldwide. Since May 2022, MPXV has spread rapidly in non-endemic mpox areas. As of 27 June 2023, the virus has spread to more than 112 countries and regions, with over 88,060 laboratory-confirmed cases and 147 deaths. Thus, measures to control the mpox epidemic are urgently needed. As the principal methods for identifying and monitoring mpox, laboratory detection techniques play an important role in mpox diagnosis. This review summarises the currently-used laboratory techniques for MPXV detection, discusses progress in improving these methods, and compares the benefits and limitations of various diagnostic detection methods. Currently, nucleic acid amplification tests, such as the polymerase chain reaction, are the most commonly used. Immunological methods have also been applied to diagnose the disease, which can help us discover new features of MPXV, improve diagnostic accuracy, track epidemic trends, and guide future prevention and control strategies, which are also vital for controlling mpox epidemics. This review provides a resource for the scientific community and should stimulate more research and development in alternative diagnostics to be applied to this and future public health crises.

## Introduction

Mpox (formerly monkeypox) is a zoonotic disease caused by monkeypox virus (MPXV), a double-stranded DNA virus belonging to the genus *Orthopoxvirus* of the family *Poxviridae* [1]. It consists of two genetic clades: clade I (formerly the Central African or Congo Basin clade) and clade II (formerly the West African clade) [2]; the latter was suggested to be the predominant subtype in the 2022 outbreak [3], with a low case fatality rate [4, 5]. In addition to MPXV, three other orthopoxviruses (OPVs), namely, smallpox virus, vaccinia virus, and cowpox virus, cause human infections [6, 7].

MPXV was first discovered in 1958 in laboratory monkeys [8]. Rodents, including African squirrels, tree squirrels, Gambian kangaroos, and dormice, are the suspected intermediate natural hosts of the virus; however, the original natural reservoir of MPXV remains unclear [9]. In 1970, the first case of human mpox was recognised in the Democratic Republic of the Congo (DRC), and human-to-human transmission was subsequently confirmed. Viral spread may occur through contact with body fluids and respiratory droplets of infected patients, the former of which is the primary mode of transmission [10, 11]. According to the United Kingdom Health and Safety Authority, in the 2022 mpox outbreak, numerous confirmed cases were identified among men who have sex with men and had no history of travel to mpox-endemic areas [12]. Their seminal fluid samples were positive for MPXV DNA, suggesting that MPXV may spread through sexual contact [11, 12].

**Fig. 1:**
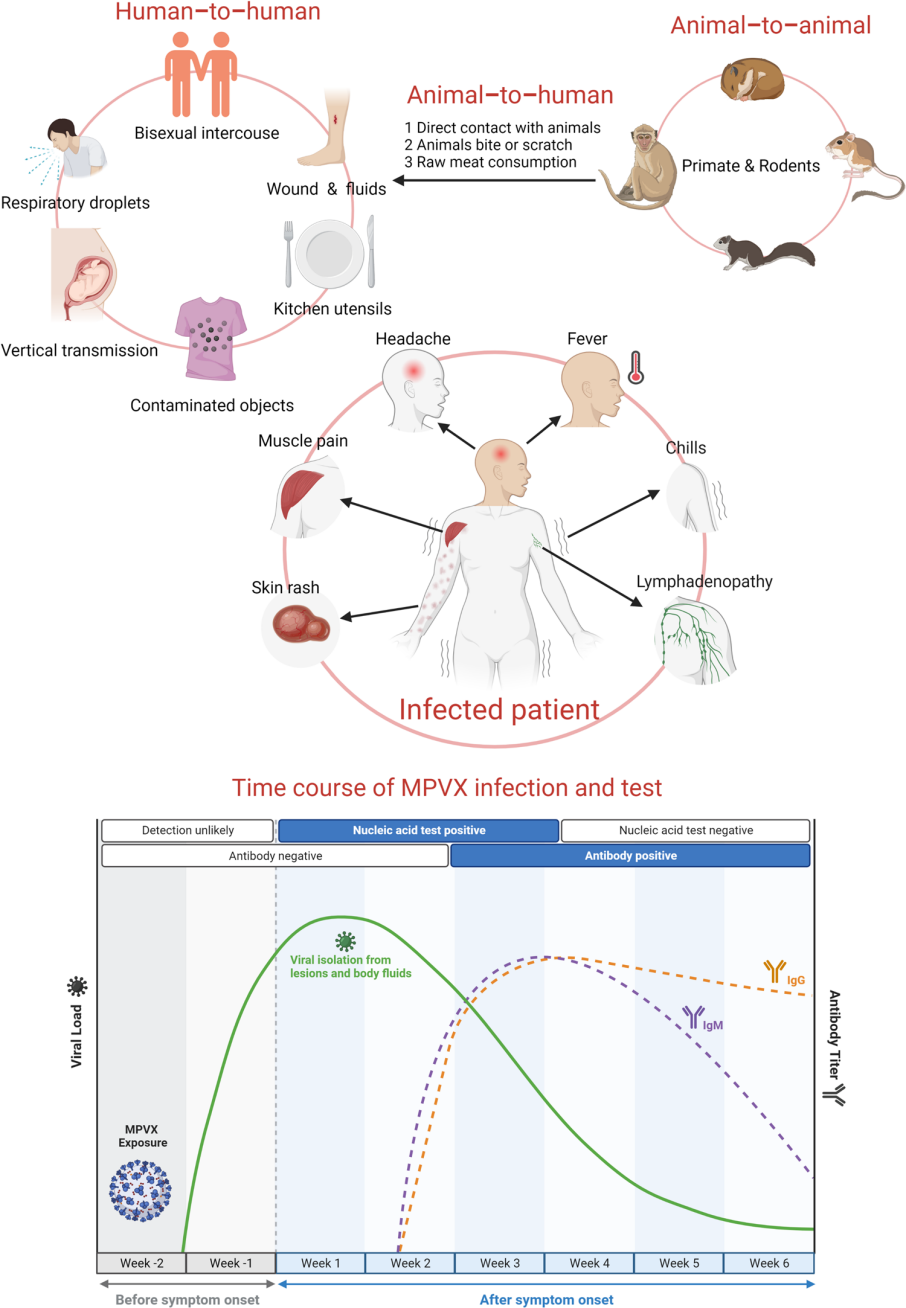
Monkeypox virus: hosts, transmission, timeline, and clinical manifestations in human organs. Created using BioRender.com

The clinical manifestations of human mpox mirror those of smallpox but are milder (Fig. 1) [13]. Mpox is a self-limiting disease characterised by skin rashes similar to those observed in smallpox, as well as lymphadenopathy, fever, chills, headache, fatigue, and other symptoms [14]. Most infected people recover within a few weeks; however, children, pregnant women, and those with a weakened immune system are at a higher risk of developing severe disease and even dying from bleeding herpes or other serious complications. Vaccines against smallpox confer 85% protection against MPXV; however, due to previous reports that MPXV was significantly less contagious than smallpox and would not persist in human communities even without vaccination, the original smallpox vaccines were discontinued [15]. To date, the following new smallpox vaccines have been suggested by the World Health Organisation (WHO) for pre- and post-exposure prophylaxis against MPXV: JYNNEOS, LC16m8, and ACAM2000 [16, 17].

**Fig. 2:**
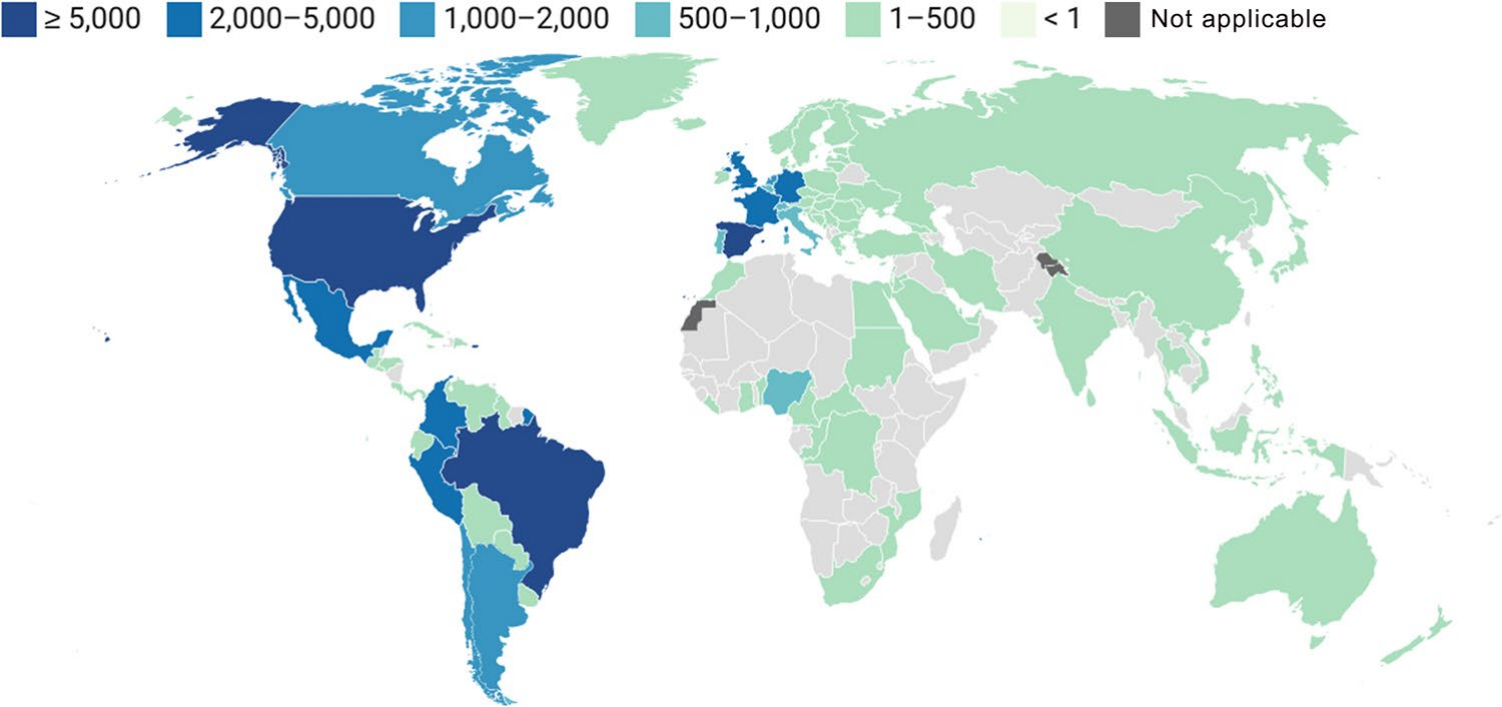
Global mpox cases in the 2022 MPXV outbreak. Countries that have reported cases of mpox are shown. The values represent the total number of cases in each country between May 2022 and June 2023. Drawn based on data from the World Health Organization [22], using Datawrapper

Mpox has long been considered an endemic disease, and its prevalence is well known in Central and West Africa, including the DRC, Central African Republic, Nigeria, and Cameroon [5]. In the recent outbreak, the first cases were reported in the United Kingdom on 7 May 2022, after which many non-endemic areas, including the Netherlands, Italy, and Brazil, confirmed their first MPXV cases [18–20]. Subsequently, the disease spread worldwide, with the number of infections rapidly increasing [5]. Consequently, in July 2022, WHO declared the global mpox outbreak a public health emergency of international concern [21]. As of 27 June 2023, MPXV has spread to more than 112 areas, with over 88,060 laboratory-confirmed cases and 147 deaths (Fig. 2) [22]. The Americas are the regions most affected by this epidemic, with approximately 59,514 cases and 117 deaths, followed by Europe, with 25,914 cases and seven deaths [22]. Although the latest report from WHO indicates a decline in the number of cases, caution should not be abandoned, and diagnostics should be improved.

Notably, genetic data showed that the currently prevalent MPXV strain is likely a descendant of the strain that caused the 2018-2019 Nigerian mpox outbreak [3]. However, the former has a 6- to 12-fold higher mutation rate than expected when compared to the natural mutation rate, especially for OPVs, which usually undergo only one or two mutations per year. Furthermore, Isidro et al. [3] demonstrated that apolipoprotein B mRNA-editing enzyme catalytic polypeptide-like 3 (APOBEC3) could alter the viral genome, mediating the microevolutionary emergence of MPXV to be more adapted to humans, facilitating human-to-human transmission. Therefore, the extent and severity of the current mpox epidemic may have been underestimated. Currently, there are no approved treatments for mpox, and mpox vaccines are unavailable to individuals younger than 18 years [17].

### MPXV detection techniques

Since the clinical manifestations caused by different OPVs are similar, identifying mpox based on symptoms alone is challenging [23, 24]. Therefore, early detection can help to identify infected people and alert them to take timely isolation and treatment measures, thereby reducing the spread of the virus and mitigating the impact of the outbreak [25]. To minimise transmission, there is an increasing need to develop diagnostic techniques with high sensitivity, high accuracy, and fast detection rates. To be specific, the test should be able to specifically detect MPXV but not other similar viruses, and its sensitivity should be as high as possible to ensure accurate and reliable results. Additionally, the test needs to be sensitive enough to detect miniscule amounts of MPXV (less than 1 fg) [26] and also be able to detect the virus in the early stages of infection for the effective prevention of outbreaks. After an mpox outbreak, in addition to quickly diagnosing the disease, it is also necessary to detect the causative strain of mpox. Since members of different clades may have different epidemiological characteristics, relevant epidemic prevention measures can be formulated according to these characteristics [27]. In addition, testing against viruses of related clades also facilitates vaccine development. However, the branch test can only be carried out after the causative virus is confirmed to be MPXV because, as MPXV continues to evolve, new mpox branches may appear, and if only one specific mpox clade is tested, false negative results may be obtained [3].

Currently, polymerase chain reaction (PCR) is still the most common technique used in MPXV testing. However, there are several other detection techniques, such as immunological methods, virus isolation from cell culture, etc., that can be used for MPXV detection [28].

In this article, we review the current laboratory techniques for MPXV detection, discuss recent progress in improving these methods, and compare the strengths and limitations of various diagnostic tests. This review may help medical professionals select suitable assays for different environments. Furthermore, we propose novel assays that may help researchers or medical professionals improve diagnosis and develop new diagnostic assays.

## Laboratory detection techniques

Laboratory virological methods are essential for the correct diagnosis and investigation of infection rates in populations. To date, MPXV infection has been confirmed unequivocally through the use of direct and indirect diagnostic methods. Of the direct tests, the nucleic acid amplification test (NAAT) is most commonly used to identify the deoxyribonucleic acid (DNA) sequences that make up the genetic material of the virus. In contrast, the indirect MPXV test detects the patient's immune response to viral infection. In the following sections, we describe basic information about sample collection, transport, and storage of MPXV. In addition, we summarise and explore the different testing strategies being developed or used for the diagnosis of mpox, discussing their advantages, limitations, and directions for application. We also present methods with potential for future applications that could meet current needs [29, 30].

### Biosafety in laboratory processing

Laboratories that use patient samples for mpox diagnosis should take steps to minimise the risk of laboratory transmission. These steps may include wearing appropriate personal protective equipment (PPE) with restricted access and ensuring that samples are handled only by trained professionals [31]. Non-transmissible diagnostic tests such as (NAAT) and some serological assays can be performed in a biosafety level 2 (BSL-2) laboratory and are recommended to be operated in at least a class II biological safety cabinet (BSC). However, procedures involving work with live viruses, such as virus culture or isolation, should only be performed in a laboratory equivalent to BSL-3 [32]. It is important to use standard precautions to avoid any transmission of infectious aerosols [31], since the occurrence of MPXV infections in healthcare workers has been documented worldwide. Therefore, when testing clinical samples from patients with suspected or confirmed mpox, measures should be taken to minimise the risk of laboratory transmission based on risk assessment.

### Specimen collection, transport, and storage

When collecting specimens, the same type of lesion can be placed in the same collection tube, whereas samples from different lesion types should be separated. Simultaneous collection of different types of lesions at different sites is preferable. In addition to specimens from skin lesions, oropharyngeal, anal, and rectal swabs can also be used to detect MPXV; however, the results of oropharyngeal specimens should be viewed with caution because of the limited clinical data regarding their use in mpox diagnosis [28]. Additional types of specimens may be collected for research purposes with the permission of the ethical review committee and under conditions where there is sufficient laboratory and medical expertise to collect, transport, and store the specimens [28]. These specimens may include urine, semen, or rectal or genital swabs, based on clinical signs, including the location of the lesion. Testing of ethylenediaminetetraacetic acid (EDTA)-anticoagulated whole blood may support the diagnosis of MPXV, but the sample may not contain high levels of virus, as viremia is only observed early in the infection, i.e., before the prodromal phase with skin lesions. Sample collection should be carried out by health professionals in accordance with appropriate standard operating procedures (SOPs), using appropriate personal protective equipment (PPE) [28].

Specimens should be refrigerated at 2-8 °C or frozen at -20 °C or lower within 1 h after collection and transported to the testing facility as soon as possible [28]. Proper handling and storage of samples during transport is a critical step in performing accurate diagnostic tests. All samples should be stored at -20 °C or lower if transported for more than 7 days. If the number of days since collection exceeds 60 days, long-term storage of samples at -70 °C is recommended [28]. The above storage practices are important to prevent false negative results. Several factors, such as poor specimen quality, improper handling or transport, or technical problems with the assay (e.g., failed DNA extraction), may affect the diagnostic performance and quality control of the reference laboratory.

### PCR

PCR and real-time PCR are recommended by WHO as routine laboratory tests for mpox [28]. If the clinical samples from individuals suspected of having mpox test positive via MPXV-specific PCR, mpox infection can be diagnosed. The laboratory’s recommended materials for confirming mpox are specimens from the skin lesion, for example, exudate and scabs, which should be collected at the macular stage using dry swabs and a viral transport medium. Blood specimens are recommended for routine PCR, because viremia occurs in the early stage of MPXV infection, when the symptoms are nonspecific [33].

According to WHO guidelines, viruses can be detected using traditional PCR. As early as 1995, Ropp et al. [34] found that the genome sequence encoding the haemagglutinin protein could be used as a target to identify OPVs using PCR primers specific for that region. However, since all OPVs contain haemagglutinin, this method cannot specifically implicate MPXV. In order to improve the detection of MPXV by traditional PCR, Meyer et al. [35] reported that the gene encoding the A-type inclusion body protein (ATI) can be used to distinguish MPXV from other OPVs in PCR assays. Later, Neubauer et al. [36] identified a unique 8-bp deletion in the ATI gene and demonstrated experimentally that it can be used for the specific detection of MPXV by PCR.

Compared to conventional PCR, real-time PCR has a higher detection speed, sensitivity, and specificity [37]. The cycle quantification (Cq) value obtained by adding a fluorescent-labelled probe to the assay tube and measuring the intensity of the fluorescence signal of the amplified product in real time can be used to confirm the presence of MPXV in the sample [38]. Combined with the patient's symptoms, a preliminary diagnosis of the disease can then be made. However, there are no definitive data on the minimum viral load required for humans to be considered infected by MPXV [39], and no significant relationship between the timing of clinical manifestations in patients and the Cq value for the virus in the plasma has been observed [40]. Furthermore, many laboratories have developed real-time PCR assays for MPXV based on F3L, N3R [41], B6R [42], B7R [43], E9L [42], C3L, G2R [26], F3L [44], and J7R [45] (Table 1).

**Table 1:** Examples of published assays for detection of monkeypox virus

Detection technique	Targeted viruses	Target gene	Conserved?	Primer sequences	Probe sequences	Detection limit	Sample	Equipment	Reference
**PCR**	Orthopoxvirus	HA gene	Yes	Forward: CTGATAATGTAGAAGAC Reverse: TTGTATTTACGTGGGTG	Undescribed	Undescribed	Human	Model 9600 thermal cycler	[34]
PCR	Monkeypox virus	ATI	Yes	Forward: GAGAGAATCTCTTGATAT Reverse: ATTCTAGATTGTAATC	Undescribed	Undescribed	Rodent	GeneAmp ® PCR System 9600	[36]
**Real-time PCR**	Monkeypox Congo basin-specific	C3L	Yes	Forward: TGTCTACCTGGATACAGAAAGCAA Reverse: GGCATCTCCGTTTAATACATTGAT	FAM-CCCATATATGCTAAATGTACCGGTACCGGA-BHQ1	9.46 fg (∼40.4 genomes)	Human	All platforms	[26]
	Monkeypox virus	G2R	Yes	Forward: GGAAAA TGTAAAGACAACGAATACAG Reverse: GCTATCACATAATCTGG AAGCGTA	FAM-AAGCCGTAATCTATGTTGTCTATCGTGTCC-BHQ1	0.7 fg (∼3.5 genomes)	Human		
	Monkeypox West-African-specific	G2R	Yes	Forward: CACACCGTCTCTTCCACAGA Reverse: GATACAGGTTAATTTCCACATCG	FAM-AACCCGTCGTAACCAGCAATACATTT-BHQ1	1.7 fg (∼8.2 genomes)	Human		
Real-time PCR	Monkeypox virus	F3L	Yes	Forward: CTCATTGATTTTTCGCGGGATA Reverse: GACGATACTCCTCCTCGTTGGT	6FAM-CATCAGAATCTGTAGGCCGT-MGBNFQ	11–55 fg (50–250 copies of each gene)	Rodent	LightCycler® System	[41]
		N3R	Yes	Forward: AACAACCGTCCTACAATTAAACAACA Reverse: CGCTATCGAACCATTTTTGTAGTCT	FAM-TATAACGGCGAAGAATATACT-MGBNFQ				
Real-time PCR	Eurasian orthopoxviruses (not variola or North American orthopoxviruses)	E9L-NVAR	No	Forward: TCAACTGAAAAGGCCATCTATGA Reverse: GAGTATAGAGCACTATTTCTAAATCCCA	TET-CCATGCAATATACGTACAAGATAGTAGCCAAC-QSY7	2.54 fg viral DNA (∼12.5 genomes)	Human	ABI7700® LightCycler® iCycler iQ™	[42]
	Monkeypox virus	B6R	Yes	Forward: ATTGGTCATTATTTTTGTCACAGGAACA Reverse: AATGGCGTTGACAATTATGGGTG	MGB/DarkQuencher- AGAGATTAGAAATA-FAM	10 viral copies (2 fg)		ABI7700® iCycler iQ™	
Real-time PCR	Monkeypox virus	B7R	Yes	Forward: ACGTGTTAAACAATGGGTGATG Reverse: AACATTTCCATGAATCGTAGTCC	TAMRA-TGAATGAATG CGATACTGTATGTGTGGG-BHQ2	20 copies/reaction	Human	Real-Time PCR System 7500	[43]
**Real-time PCR**	Monkeypox virus	F3L	Yes	Forward: CATCTATTATAGCATCAGCATCAGA Reverse: GATACTCCTCCTCGTTGGTCTAC	JOE-TGTAGGCCGTGTATCAGCATCCATT-BHQ1	20 copies/reaction	Rodent	Real-Time PCR System 7500	[44]
Real-time PCR	Monkeypox virus	J7R (haemagglutinin gene)	Yes	Forward: GATGATGCAACTCTATCATGTA Reverse: GTATAATTATCAAAATACAAGACGTC	FAMAGTGCTTGGTATAAGGAG MGBNFQ	50 copies/assay (10 fg)	Rhesus macaque	Undescribed	[45]
**GeneXpert**	Monkeypox virus	G2R	Yes	Forward: GGAAAA TGTAAAGACAACGAATACAG Reverse: GCTATCACATAATCTGG AAGCGTA	FAM-AAGCCGTAATCTATGTTGTCTATCGTGTCC-BHQ1	12.5 genome copies (2.56 fg)	Human	GeneXpert platform	[48]
LAMP	Monkeypox Congo-basin-specific	D14L	No	FIP: TGGGAGCATTGTAACTTATAGTTGCCCTCCTGAACACATGACA F3: TGGGTGGATTGGACCATT BIP: ATCCTCGTATCCGTTATGTCTTCCCACCTATTTGCGAATCTGTT B3: ATGGTATGGAATCCTGAGG Loop-F: GATATTCGTTGATTGGTAACTCTGG Loop-B: GTTGGATATAGATGGAGGTGATTGG	Undescribed	10^2.4^ copies/reaction	Vero and HeLa cell	Loopamp® DNA Amplification Kit	[53]
	Monkeypox-West African-specific	ATI	Yes	FIP: CCGTTACCGTTTTTACAATCGTTAATCAATGCTGATATGGAAAAGAGA F3: TACAGTTGAACGACTGCG BIP: ATAGGCTAAAGACTAGAATCAGGGATTCTGATTCATCCTTTGAGAAG B3: AGTTCAGTTTTATATGCCGAAT Loop-F: GATGTCTATCAAGATCCATGATTCT Loop-B: TCTTGAACGATCGCTAGAGA	Undescribed	10^3^ copies/reaction			
	Monkeypox virus	ATI	Yes	FIP: TGGAGTCTGCTAATCTCTGTAAGATTAGAGAACTAGAGAATAAGTTGACC F3: CACAAGAAGTTGATGCACTG BIP: TGAGTGAATGCCGTGGAAATGCGCAGTCGTTCAACTGTA B3: CAGCATTGATTTCATTATTACGT Loop-F: CGCTCTCGATGCAGTC Loop-B: CAGAGATTACAATCTAGAATCTCAG	Undescribed	10^2^ copies/reaction			
RPA	Monkeypox virus	G2R	Yes	Forward: AATAAACGGAAGAGATATAGCACCACATGCAC Reverse: GTGAGATGTAAAGGTATCCGAACCACACG	ACAGAAGCCGTAATCTATGTTGTCTATCGQTFCCTCCGGGAACTTA	16 DNA molecules/µL	Human	ESEQuant	[57]

The assays that have been approved by the WHO interim guidance for detecting monkeypox virus infections are marked in boldfaced font.

Many laboratories have begun to optimise the detection capabilities of the real-time PCR platform. Chelsky et al. [46] developed a nucleic-acid-extraction-free approach to PCR to improve the scalability of MPXV testing. This method solved, to some extent, the problems of non-standard nucleic acid extraction procedures and a shortage of extraction kits. This assay also reduced the processing time per sample and decreased exposure to contaminants while retaining the accuracy and sensitivity of the original PCR assay, making it easier to develop large-scale testing for MPXV. In September 2022, the Quest Diagnostics Monkeypox Virus DNA, Qualitative, Real-Time PCR test kit (San Juan Capistrano, California) (“Quest Monkeypox PCR, Test code: 12084”) received emergency use authorisation from the FDA to detect the presence of MPXV DNA in lesions from individuals suspected of having mpox. This was the first MPXV test kit authorised by the FDA, providing timely and effective support for the prevention and control of the mpox outbreak [47]. In addition, Li et al. [48] developed the Cepheid GeneXpert system, a backpack-sized analytic workstation that combined sample preparation, real-time PCR amplification, and MPXV detection. This system is site-independent and allows PCR testing to be performed anytime and anywhere.

### Isothermal amplification techniques

Isothermal amplification is an *in vitro* nucleic acid amplification technique in which rapid amplification of nucleic acids is achieved by adding enzymes and specific primers at a constant temperature [49]. In contrast to PCR techniques, this method does not require large or expensive thermocyclers and can be performed under simple conditions at a constant temperature [50], extending its application to resource-limited settings, such as temporary medical sites. Its rapid, efficient, and specific characteristics improve the accuracy and sensitivity of on-site detection of viral nucleic acids. Currently, the main isothermal amplification techniques reported for MPXV detection include loop-mediated isothermal amplification (LAMP) and recombinase polymerase amplification (RPA) [49].

LAMP is a novel thermostatic nucleic acid amplification method. At 60-65 °C and in the presence of *Bst* DNA polymerase, four to six primers specific for six characteristic regions of the virus target gene can be used to achieve rapid amplification of the desired regions in as little as 1 h [51]. Feng et al. [52] utilised conserved regions of the A27L and F3L genes of MPXV as target sequences to design specific primers. They found that A27L-1 and F3L-1 initiated the fastest and most sensitive LAMP reaction among all of the primers tested, with an accuracy of approximately 100 times higher than that of the conventional PCR method, providing an effective target for clinical detection of MPXV. However, given that the genetic material being detected was in an artificial model rather than a natural virus, the generalisability of the experimental results requires further investigation [44, 52]. Iizuka et al. [53] identified the target genes of the two major MPXV clades: the D14L gene of clade I and part of the ATI gene of clade II. This finding will allow accurate differentiation between the two clades and will help in the epidemiological assessment of the MPXV infection route. However, designing the correct set of LAMP primers can be challenging. The primers must recognise six independent sequences on the target DNA/RNA [54]. This requires optimising the primers to bind at separate target sites. The regions targeted by the primers must be very close together, within 2-3 base pairs. However, sites that are too close may interfere with each other. So designing primers is more complicated in this method compared to PCR, with an increased risk of nonspecific amplification and false-positive results [49].

Compared to LAMP, RPA has a faster reaction speed and can achieve the same level of amplification of the target gene in a shorter time [55]. This technique is performed at 37-43 °C with three main proteins: recombinase, recombinase loading factor, and single-stranded binding protein [55, 56]. Davi et al. [57] confirmed that the results obtained from the detection of the tumour necrosis factor (TNF)-binding protein gene of MPXV by RPA are consistent with those obtained by conventional PCR, indicating that RPA may be a useful technique for the clinical detection of MPXV nucleic acids. However, RPA detection is not sufficiently developed, and nonspecific amplification occurs frequently. The biggest problem in mpox detection is a lack of primer or probe specificity. Since the primers or probes are not fully optimised to uniquely amplify the target sequence, multiple sequences can be amplified nonspecifically, leading to errors in RPA results [58]. Considering the advantages of rapid amplification, high sensitivity, and compatibility with multiplexing, recombinant-enzyme-based methods (RPA/RAA) have the potential to create field diagnostics suitable for resource-limited settings.

### Clustered regularly interspaced short palindromic repeats (CRISPR)

CRISPRs are repetitive sequences in prokaryotic genomes. Cas genes are associated with CRISPR arrays and usually contain a nuclease for nucleic acid cleavage that can specifically identify and cleave target DNA [59]. The CRISPR-Cas system is an acquired immune system in prokaryotes that defends against foreign nucleic acid invasion and has been applied in viral nucleic acid detection methods; this system has been widely used in clinical and scientific studies [60]. Cas12 is an RNA-guided enzyme that cleaves targeted DNA [61]. The CRISPR/Cas technique is an promising molecular detection technique that relies on the Cas12 protein for precise cleavage of MPXV DNA [62]. MPXV can be detected using a CRISPR-Cas12-based reverse-transcriptase-mediated isothermal amplification approach [63]. Using the F3L and N3R genes as reporter genes, Sui et al. [64] demonstrated that the CRISPR-Cas12-based MPXV detection method utilizing fluorescent readout can be used to measure a statistically significant nucleic acid fluorescence signal despite low virus titres. Mao et al. [63] and Chen et al. [62] succeeded in specifically detecting MPXV, without cross-reactivity with other OPVs, using RPA combined with CRISPR-Cas detection. Methods based on CRISPR-Cas12 provide more convenient and reliable options for the rapid detection of MPXV, thus improving the sensitivity and specificity of the detection of infected (including asymptomatic) individuals and interrupting viral transmission rapidly and effectively.

### Immunological methods

Because OPVs are immunologically cross-reactive [65], none of the tests associated with antigens and antibodies are sufficiently specific to diagnose mpox and are prone to providing false-positive results [65]. In addition, immunoassay specimens have complex storage and transportation requirements; moreover, these assays are not as rapid or accurate as molecular assays [30]. These shortcomings limit the widespread use of immunological methods for MPXV; nevertheless, they are still useful for studying the epidemiology of outbreaks and epidemics in prevalent areas where resources for viral nucleic acid testing are limited [30]. Immunological detection techniques for MPXV include immunohistochemistry, enzyme-linked immunosorbent assay (ELISA), western blot (WB), and radioimmunoassay (RIA), all of which are based on the principle of specific binding of antigens to antibodies.

The principles of immunohistochemistry and ELISA are similar, the former detects viral antigens in tissues or cells, whereas the latter detects IgG and IgM antibodies [66]. Using polyclonal or monoclonal antibodies specific for all OPVs, immunohistochemistry can be used to distinguish between poxviruses and herpesviruses [66]. The MPXV A29 protein is the envelope protein of the virus, which mediates virus recognition by the host cell and is considered the most important protein target for MPXV immunoassays [67]. ELISA is the preferred method for serum antibody detection; specific IgM and IgG antibodies can be detected 7 or 21 days after the onset of rash in infected individuals [68]. MPXV infection can be diagnosed if the IgG titre in the recovery phase is at least four times higher than that in the acute phase [68]. However, the results of ELISA may be influenced by smallpox vaccination [61] due to the low specificity of IgM and IgG detection of MPXV. In 2008, Dubois et al. [69] proposed a pre-absorption step before ELISA to deplete as many cross-reactive antibodies as possible. They added 6 × 10^8^ FPU of inactivated MPXV or vaccinia whole-cell lysate per mL to the plasma samples (30:1), thereby enabling differentiation between vaccinia virus and MPXV. In a later study, they demonstrated that an ELISA for MPXV based on the MPXV B21R protein peptide segment had high sensitivity (100%) and specificity (92%) at 2-6 months postinfection, and this technique has since been used in retrospective MPXV studies [70]. Moreover, Ichihashi et al. [71] showed that using the competitive binding inhibition assay method, the antibodies in the sera of MPXV-infected individuals bound MPXV competitively with the MPXV-specific monoclonal antibody (MAb) H12C1 but did not affect the binding of vaccinia virus to the vaccinia-specific MAb G6C6. Antibodies in the sera of both vaccinated and naturally MPXV-infected individuals compete for both MAbs. This method makes it possible to differentiate between individuals who are naturally infected with MPXV and previously smallpox-vaccinated patients with MPXV infection [71].

JOYSBIO [72] has successfully developed a rapid test kit that employs a lateral flow immunoassay cassette for the qualitative detection of MPXV antigens and antibodies during infection. Antigens can be detected by collecting skin lesions and related infected tissues. However, to use this kit, a drop of blood needs to be drawn from the subject for testing, and special attention needs to be paid to avoid biological contamination. The advantage of this rapid detection kit is that it can provide results rapidly (15 min). Compared with other detection methods, the kit is very simple to operate, and medical staff can easily collect test specimens from patients' skin lesions. Potential limitations of this method include low sensitivity and erroneous results, as well as the need for careful waste disposal.

Additional techniques based on the same principle as that of other immunoassays include WB, RIA, and haemagglutination inhibition (HI) tests, and these can also be used for the detection of MPXV. WB is a technique that allows detection of specific MPXV proteins in complex samples [73]. HI is a serological assay used to detect viruses that have a haemagglutinin protein on their surface, allowing them to agglutinate red blood cells. Since MPXV particles contain haemagglutinin, inhibition of erythrocyte adhesion and aggregation by antibodies in the serum of the patient can aid in the diagnosis of an MPXV infection. RIA is an ultramicroscopic analytical technique that uses both labelled isotopes of antigens and unlabelled antigens to competitively bind specific antibodies simultaneously for quantitative detection of MPXV antigens in the specimen [74]. However, they are not widely used for MPXV detection due to practical limitations (Table 2) and are mainly used in retrospective studies of mpox epidemics.

**Table 2:** Summary of monkeypox virus detection methods

	Time	Sample sources	Advantages and disadvantages	Application scenarios
PCR	3-5 h	Clinical samples^; virus isolates; synthetic templates and laboratory-inoculated samples	Sensitivity and specificity; inexpensive	Virus detection;
Real-time PCR	1-3 h	Clinical samples^; virus isolates; synthetic templates and laboratory-inoculated samples	High sensitivity and specificity; rapidness; applicable in various platforms; inexpensive	Virus detection; viral load evaluation
Extraction-free PCR	30-60 min faster than PCR detection	Clinical samples^ (samples with blood contamination are not recommended)	High sensitivity and specificity; rapidness; no need to extract nucleic acid; sample requirements are high	Suited for the field and point-of-care
GeneXpert	About 1 h	Clinical samples^; virus isolates; synthetic templates and laboratory-inoculated samples	Reduced contamination; minimised sample amount	Suited for the field and point-of-care
LAMP	30-60 min	Clinical samples^; virus isolates; synthetic templates and laboratory-inoculated samples	No need for a light cycler; less time; no need for a complex thermocycler	Type identification; survey the course of infection
RPA	5-15 min	Clinical samples^; virus isolates; synthetic templates and laboratory-inoculated samples	No need for a complex thermocycler; reaction performs in low temperature for short time	Suited for the field and point-of-care
CRISPR	About 35 min	Clinical samples^; virus isolates; synthetic templates and laboratory-inoculated samples	Technology research in progress	Not routinely used
ELISA	3-4 h	Whole blood or serum	Incapable of type differentiation; cross-reactivity among orthopoxviruses	IgM reacts to recent infections; IgG reacts to distant infections
WB	3-4 h	Virus isolates; virus extraction solution	Sample requirements are high; limited sample throughput	Not routinely used
RIA	24 h or more	Whole blood or serum	High sensitivity and specificity; radioactivity; expensive	Not routinely used
HI	Approximately 30-60 min	Serum	Simple and inexpensive; requires fresh red blood cells; not sufficiently specific	Epidemiological studies;
Electron microscopy	1 week	Virus isolates	Detection of viruses even at early stages; expensive; trained staff; well-equipped laboratories; incapable of type differentiation	Visualisation of morphological features
Virus isolation and culture	1-4 days or more	Focal biopsy samples and laboratory-inoculated samples	Expensive; trained staff; BSL3 level lab; time-consuming	Viral pathobiology studies; vaccine development research
Whole-genome sequencing	5-10 days	Virus isolates and laboratory-inoculated samples	Expensive; time-consuming	Virus traceability; virus mutation analysis

^Preferred types: swabs of lesion surface or exudate, roofs from multiple lesions, or lesion crusts

Additional types: semen, urine, rectal, or genital swabs or venous whole blood collected in EDTA

### Electron microscopy (EM)

EM is an important tool that is frequently used to study the ultrastructure of viruses and is sometimes used for viral diagnosis [75]. It is the most direct method for virus observation and detection and can be used to identify virus particles in rashes, blister fluid, and scabs [76]. Under an electron microscope, MPXV resembles OPVs in size and morphology, with oval or brick-shaped particles approximately 200-300 nm in size [77]. However, observation of such particles only suggests that the virus belongs to the genus *Orthopoxvirus* but does not identify the precise species [78]. Moreover, sample preparation for EM is time-consuming and complicated, requiring specialised knowledge to perform microscopic observation [78]. There is also a risk of infection for laboratory personnel when isolating MPXV. Therefore, this method is not suitable for large-scale use and is often combined with other detection techniques, such as molecular and immunological detection methods, to improve the sensitivity of virus detection [75].

### Virus isolation and culture

Virus isolation and culture are classical methods for the diagnosis of viral diseases. The ability to isolate and culture MPXV in a lab environment is fundamental to its study and management. Isolated viruses can be characterised in depth through sequencing and used for testing of antivirals, the development of medical countermeasures such as vaccines, and the development of research techniques and clinical applications. Outbreak investigation and containment frequently rely on isolating viruses from key cases to determine their origin, identify mutations, and reconstruct transmission events by comparing genomic sequences and phenotypes among isolates.

MPXV grows well in mammalian cells and tissues, including the cell lines HeLa, Vero, BSC-1, and RK-13, and chicken embryos are also sensitive to poxviruses [79–82]. MPXV can grow well and cause cytopathic lesions in the chorioallantoic membranes (CAMs) of chicken embryos. After 1-4 days following virus inoculation, rounding and granulation of CAM cells, cytoplasmic bridging, and syncytium formation can be observed using EM [83]. However, typical rounded and detached cells may be observed after a much shorter time period (~24 h) when MPXV is cultured in Vero cells rather than CAMs, and the virus particles can subsequently be identified using immunofluorescence and specific antibodies [82]. Although the results of this method are accurate, the time required for detection is long. In addition, performing MPXV isolation and culture requires a high level of laboratory biosafety (level 3 or higher) and experienced personnel, and infection may still occur even with complete personal protection [28]. These issues significantly limit the widespread use of this method.

### Whole-genome sequencing (WGS)

WGS is a next-generation sequencing technique by which the entire genome of an organism is sequenced, and this is the most accurate method for distinguishing MPXV from other OPVs [84]. It covers a wider range of pathogens than other molecular diagnostic techniques, and it permits comprehensive bioinformatic analysis, aiding in the development of detailed virological analyses and associated immunoassays to further advance the study of viruses. It allows the identification of specific strains and genetic variants and can be used to infer the origin of an outbreak, especially when the chain of transmission is unknown [85]. In addition, WGS data can be used to trace genetic changes that have accumulated over time, providing insight into how the virus has adapted to various ecological niches, hosts, and public health measures. WGS is an important way to identify genetic markers of antiviral resistance or severe disease, allowing important mutations to be monitored [84], and it can even facilitate the early detection of epidemiologically significant variants that are potential future pandemic threats. The availability of a large number of viral genome sequences also allows high-resolution mapping of mpox phylogeny and biogeography. By comparing many genome sequences from outbreaks across regions, migration patterns of the virus can be inferred. This technique is becoming more widely recognised as a powerful tool for epidemiological studies, and its findings are useful for disease treatment and vaccine development, providing a scientific basis for precise prevention and control of mpox outbreaks. However, because it requires considerable computational power for sequencing data storage and processing and has a high operational cost, WGS is not suitable for large-scale testing. The feasibility and implementation of WGS also depends on overcoming considerable practical, ethical, and scientific limitations through continued development, coordination, and thoughtful policymaking. While it is not a feasible point-of-care test, its results can be applied for the development of other diagnostics [85]. At present, the utility of WGS-based detection is mainly evident in research and some case reports, and an MPXV database has also been established based on these studies. WHO strongly recommends that national and molecular laboratories working on mpox diagnostics contribute their information to existing databases [86].

## Conclusion and future directions

Nucleic acid amplification-based diagnosis was introduced as the gold standard method for detecting MPXV and is able to differentiate between different virus types. This method operates on a variety of platforms, with real-time PCR technology being the most effective for diagnosing and typing MPXV. High sensitivity, specificity, rapidity, validity, and high throughput make this method preferable to others. Although methods such as electron microscopy and cell culture are not suitable for routine diagnosis, they can be useful for basic research in molecular pathobiology and the development of vaccine delivery methods, but they require well-equipped laboratories and well-trained personnel. In addition, the development of a rapid diagnostic platform for MPXV antigen detection for screening tests could enable rapid detection and preventive measures in endemic and non-endemic areas.

Additional tests not discussed in this review, such as virus neutralisation [87], gel precipitation [88], indirect fluorescent antibody tests [89], and complement fixation tests [88], are not routine laboratory tests for MPXV, but they also have some diagnostic value.

Each detection method has its strengths and limitations; thus, it remains challenging to meet the needs of all testing situations. However, combining methods may compensate for the low sensitivity or specificity of a single test and improve the rate of early detection of MPXV as well as diagnostic accuracy.

In addition, several promising strategies, such as the use of wearable devices [90], artificial intelligence [91], and biosensors [92] have been applied to the detection of MPXV. With the support of artificial intelligence and other technologies, the sensitivity and specificity of existing techniques can be effectively improved [91]. The development of new techniques may provide more effective ways of determining the sources of infection, detecting cases, and controlling epidemics [93]. Moreover, we need not only to improve the techniques for identification of MPXV-infected individuals but also to intercept and prevent the spread of the virus as early as possible, which also requires the use of laboratory tests.

Increasing attention should be paid to MPXV. Strengthening epidemiological surveillance and improving existing or developing new laboratory tests are fundamental for protecting people’s lives and health.

## Data Availability

Publicly available datasets were analysed in this study. The data used can be found at https://worldhealthorg.shinyapps.io/mpx_global/.
